# Protocol update for the SABATO trial: a randomized controlled trial to assess early oral switch therapy in low-risk *Staphylococcus aureus* bloodstream infection

**DOI:** 10.1186/s13063-020-4102-0

**Published:** 2020-02-12

**Authors:** Achim J. Kaasch, Anna Rommerskirchen, Martin Hellmich, Gerd Fätkenheuer, Reinhild Prinz-Langenohl, Siegbert Rieg, Winfried V. Kern, Harald Seifert, for the SABATO trial group

**Affiliations:** 1 Institute of Medical Microbiology and Hospital Hygiene, Faculty of Medicine, Heinrich Heine University Düsseldorf, Düsseldorf, Germany; 2 Institute of Medical Microbiology and Hospital Hygiene, Faculty of Medicine, Otto-von-Guericke University, Leipziger Str. 44, 39120 Magdeburg, Germany; 3 Institute of Medical Statistics and Computational Biology, Faculty of Medicine, University of Cologne, Cologne, Germany; 4 Division of Infectious Diseases, Department I of Internal Medicine, University Hospital of Cologne, Cologne, Germany; 5 German Centre for Infection Research (DZIF), partner site Bonn-Cologne, Cologne, Germany; 6 Clinical Trial Center Cologne, University of Cologne, 50935 Cologne, Germany; 7 Division of Infectious Diseases, Department of Medicine II, Medical Center, University of Freiburg, Faculty of Medicine, University of Freiburg, 79106 Freiburg, Germany; 8 Institute for Medical Microbiology, Immunology and Hygiene, University of Cologne, Cologne, Germany

**Keywords:** Oral switch therapy, Intravenous, Antimicrobial, Bloodstream infection, Bacteremia, *Staphylococcus aureus*, Randomized controlled trial, Pragmatic trial

## Abstract

**Background:**

SABATO (*Staphylococcus aureus* bacteremia antibiotic treatment options) is a randomized, parallel-group, clinical non-inferiority trial designed to examine the efficacy and safety of early oral switch therapy in low-risk *Staphylococcus aureus* infection. The original trial protocol was published in *Trials* (accessible at 10.1186/s13063-015-0973-x). Here we describe final amendments to the study protocol and discuss the underlying rationale.

**Methods/design:**

Three major changes were introduced into the study protocol: (1) the inclusion and exclusion criteria were refined so that patients with certain comorbidities (end-stage renal disease, severe liver disease) and uninfected foreign bodies (orthopedic prosthesis, pacemaker, implanted cardiac cardioverter-defibrillator) became eligible for enrollment under certain conditions; (2) the target sample size was decreased by choosing a conventional non-inferiority margin of 10% and converting the interim analysis (215 patients) into the final analysis; and (3) an additional follow-up visit after 30 days was introduced to allow for a closer follow-up of patients.

**Conclusion:**

Changes to the study protocol were introduced to improve the enrollment and follow-up of patients. Furthermore, the decrease of the sample size will facilitate completion of the trial.

**Trial registration:**

ClinicalTrials.gov, NCT01792804. Registered on 13 February 2013.

German Clinical trials register, DRKS00004741. Registered on 4 October 2013, EudraCT 2013-000577-77

## Background

*Staphylococcus aureus* bloodstream infection (SAB) is a frequent and devastating disease with crude mortality of 20–30% after 30 days [1]. Despite the frequency of the infection, there remain many open questions about antimicrobial treatment. One controversy is whether antimicrobial therapy can be delivered via the oral route instead of the standard practice of intravenous treatment. We designed the *Staphylococcus aureus* bacteremia antibiotic treatment options (SABATO) study as a randomized, parallel-group, clinical non-inferiority trial to examine the efficacy and safety of early oral switch therapy in low-risk *Staphylococcus aureus* infection. The full study protocol was published previously [2].

The SABATO study population comprises patients with SAB that have a low risk of SAB-related complications. These patients are usually treated with intravenous antimicrobial therapy for 14 days. The intervention consists of a switch to oral medication after 5–7 days of intravenous antimicrobial therapy. The primary endpoint in the SABATO study is the rate of SAB-related complications (relapsing SAB, deep-seated *S. aureus* infection, or attributable mortality) within 90 days. Secondary endpoints include mortality, length of hospitalization, and complications of intravenous therapy.

Slow enrollment into the study led to a number of changes to the study protocol. Here we present the major changes and discuss the underlying rationale.

## Methods/design

Three major changes were implemented in the study protocol. First, the inclusion and exclusion criteria were refined to increase the number of eligible patients. Thus, patients with end-stage renal disease, severe liver disease, and uninfected foreign bodies (orthopedic prosthesis, pacemaker, implanted cardiac converter-defibrillator) became eligible for enrollment. Second, the target sample size was decreased and the previous interim analysis in 215 patients was converted to the final analysis. This accommodates a conventional non-inferiority margin of 10% (previously 5%). Third, an additional follow-up visit was introduced at day 30 to monitor patients more closely after the end of treatment. All changes were endorsed by the SABATO Scientific Advisory Board.

### Changes to the inclusion and exclusion criteria

Patients with prosthetic devices, end-stage renal disease, or severe liver disease were excluded in the first version of the trial protocol. However, the prevalence of these conditions has increased, so that a change seemed appropriate to improve the generalizability of the trial results.

Patients with end-stage renal disease and severe liver disease carry an increased risk of SAB-related complications [3, 4]. However, it is unclear whether SAB-related complications are also more frequent in patients with low-risk foci. An analysis from our INSTINCT (INvasive *Staphylococcus aureus* INfection CohorT) dataset showed that patients with end-stage renal disease or severe liver disease did not have increased risk of a SAB-related complication when eligibility was restricted to patients with catheter-related infection or skin-soft-tissue infection (unpublished). Thus, we considered it safe to enroll patient with this condition.

Furthermore, patients with end-stage renal disease undergoing hemodialysis have a greater risk of infective endocarditis than the general population [5]. Patients with infective endocarditis, as those with other deep-seated foci of infection, were excluded from the SABATO study. Echocardiography is mandatory before enrolling patients with end-stage renal disease, to prevent accidental enrollment of patients with infective endocarditis into the study.

In patients with (presumably) uninfected foreign bodies, an early oral switch regimen holds two specific risks. First, the prosthetic device may be falsely classified as uninfected and then extended antibiotic treatment and device removal would be necessary. Second, if oral therapy turns out to be less effective, patients on oral therapy may suffer hematogenous seeding to the prosthetic device. In this case, longer antimicrobial treatment and removal of the device would be necessary. The first risk (misclassification) was addressed by adding additional conditions, i.e. (1) a primary, low-risk infective focus needed to be established; (2) a device-associated infection needed to be ruled out by imaging; and (3) in the presence of a pacemaker or automatic implantable cardioverter-defibrillator (AICD), echocardiography needed to be performed to rule out device-associated infection. The second risk (hematogenous seeding) was addressed by only allowing enrollment of patients with devices that have been implanted more than 6 months prior, which have a reduced likelihood of infection. We further conducted a detailed analysis of patients enrolled in the INSTINCT cohort and found that the presence of pacemakers and AICD did not influence the rate of SAB-related events at 90 days [6].

A further change concerned the requirement for negative follow-up blood cultures. We realized that follow-up blood samples were not reliably obtained in the first 72 h, especially in patients with very few apparent symptoms of SAB. Therefore, the time window was increased to 96 h to account for variations in daily clinical practice and increase the pool of eligible patients.

### Change in target sample size

When designing the study, considerable debate went into defining the appropriate non-inferiority margin. A conventional 10% margin was considered attainable, but seemed too high for the clinical question to be addressed. Therefore, we chose a 5% margin, which yielded a total sample size of 430 patients (one-sided α = 0.05, β = 0.2, one interim analysis at information fraction 0.5 using the O’Brien-Fleming bound 2.373) [2]. Nota bene, to accommodate conflicting views about the size of the non-inferiority margin we planned a sequentially rejective testing procedure, i.e. (1) starting with a margin of 10% at a one-sided level of 5%, followed by (2) testing with the same margin at the stricter (and more conventional) one-sided level of 2.5%, followed by (3) testing with a margin of 5% at a one-sided level of 5%, followed by (4) testing the same margin at a one-sided level of 2.5%. However, slow enrollment necessitated a decrease in the target sample size. We thus chose to convert the scheduled interim analysis based on 215 patients to the final analysis. This accommodated a non-inferiority margin of 10% while keeping all other parameters constant. The amended study design should still be able to give convincing evidence from tests (1) and (2) (see above).

The sample size for a 10% non-inferiority margin without employing an interim analysis was calculated as follows: assuming 2.5% complications per arm, a non-inferiority margin of 10%, one-sided alpha of 2.5% and power of 80% requires 144 subjects in total (calculated using R version 3.3.3, package gsDesign, function nBinomial; R Foundation for Statistical Computing, Vienna, Austria), i.e. 144/0.9/0.9/0.95 = 187 subjects, adjusted for deaths unrelated to SAB (10%), for protocol violations (10%) and for stratification (5%). Observing 2.5% SAB-related complications in the final analysis (with 215*0.9*0.9*0.95/2 ≈ 83 patients per group), i.e. 0.025*83 ≈ 2 SAB-related complications in each group, yields a 90% confidence interval for the difference of − 0.049 to + 0.049 (Stata 15.1, StataCorp LLC, College Station, TX, USA; rdcii).

### Additional follow-up visit

At 90 days after the first positive blood culture, a follow-up visit is conducted to assess patient outcome either as an in-person visit or a telephone call. Patients are asked to report adverse events that occurred during the follow-up period to the study center. However, not all patients reported adverse events in a timely way. Thus, we added an additional visit 30 days after the first positive blood culture to minimize the potential risks to the patients and to reduce reporting bias.

## Conclusion

Due to slow enrollment of patients the protocol of the SABATO study underwent major changes. The number of eligible patients was increased by refining the inclusion and exclusion criteria whilst the target sample size was decreased by choosing a conventional non-inferiority margin of 10%. Thus, completion of the trial is facilitated.

## Data Availability

Not applicable.
